# The interplay between the microbiota, diet and T regulatory cells in the preservation of the gut barrier in inflammatory bowel disease

**DOI:** 10.3389/fmicb.2023.1291724

**Published:** 2023-12-01

**Authors:** Kathryn Prame Kumar, Joshua D. Ooi, Rimma Goldberg

**Affiliations:** Centre for Inflammatory Diseases, Department of Medicine, School of Clinical Sciences at Monash Health, Monash Medical Centre, Monash University, Clayton, VIC, Australia

**Keywords:** inflammatory bowel disease, T regulatory cells, gut permeability, diet, microbiota

## Abstract

Inflammatory bowel disease (IBD) is becoming more common in the Western world due to changes in diet-related microbial dysbiosis, genetics and lifestyle. Incidences of gut permeability can predate IBD and continued gut barrier disruptions increase the exposure of bacterial antigens to the immune system thereby perpetuating chronic inflammation. Currently, most of the approved IBD therapies target individual pro-inflammatory cytokines and pathways. However, they fail in approximately 50% of patients due to their inability to overcome the redundant pro inflammatory immune responses. There is increasing interest in the therapeutic potential of T regulatory cells (Tregs) in inflammatory conditions due to their widespread capability to dampen inflammation, promote tolerance of intestinal bacteria, facilitate healing of the mucosal barrier and ability to be engineered for more targeted therapy. Intestinal Treg populations are inherently shaped by dietary molecules and gut microbiota-derived metabolites. Thus, understanding how these molecules influence Treg-mediated preservation of the intestinal barrier will provide insights into immune tolerance-mediated mucosal homeostasis. This review comprehensively explores the interplay between diet, gut microbiota, and immune system in influencing the intestinal barrier function to attenuate the progression of colitis.

## Inflammatory bowel disease

Inflammatory bowel disease (IBD) represents a chronic inflammatory disorder affecting the gastrointestinal tract. The etiology of IBD involves a complex interplay between genetic susceptibility, environmental factors, and dysregulated immune responses (Baumgart and Carding, 2007). Notably, increased intestinal permeability is a latent condition implicated in various disorders such as type 1 diabetes, multiple sclerosis and rheumatoid arthritis (Kinashi and Hase, 2021). In IBD, there are cases of increased gut permeability in patients, occurring alongside incidences of microbial dysbiosis, and chronic inflammation (Hollander et al., 1986; Hollander, 1988; Adenis et al., 1992; Söderholm et al., 1999; Gitter et al., 2001; Tamboli et al., 2004; Pochard et al., 2018). The compromised integrity of the mucosal barrier permits bacterial substances to translocate into the underlying tissues, subsequently triggering the immune system. Excessive production of inflammatory mediators exerts direct detrimental effects on the intestinal barrier by propagating cellular apoptosis, erosions, and ulcerations in IBD (Kucharzik et al., 2001; Heller et al., 2005; Hansberry et al., 2017; Vespa et al., 2022). The management of IBD typically involves a short course of biologics and small molecules with corticosteroids or long term administration of aminosalicylates (Gomollón et al., 2017). However, a significant proportion of patients experience adverse effects or develop non-responsiveness, necessitating the consideration of surgical interventions with the percentage of patients needing surgery over the course of 1, 5, and 10 years being 4, 8.8, and 13.3% for patients with ulcerative colitis (UC), and 18.7, 28.0, and 39.5% for patients with Crohn's disease (CD), respectively (Tsai et al., 2021). The emergence of biologic therapies in clinical practice has provided more targeted and effective immunomodulation, of which the most common are tumor necrosis factor (TNF)-α blockers such as infliximab and adalimumab (Rawla et al., 2018). These biologics have been designed to suppress a hyperactive immune system, yet up to 30% of patients fail a biologic due to resistance to treatment or complications (Papamichael et al., 2016; Moss, 2022). Exclusive enteral nutrition and partial enteral nutrition are established therapies in IBD (Yamamoto and Shimoyama, 2023). Therefore, knowing more about the mechanisms of how dietary therapies manipulate inflammation in IBD can facilitate the development of targeted and safer therapies.

There is the question of whether IBD-related mucosal permeability is a primary event or a consequence of inflammation. Notably, instances of intestinal hyperpermeability may precede the onset of IBD, as suggested by a prospective study indicating that abnormal gut permeability and a disorganized gut barrier heighten the susceptibility to developing CD (Turpin et al., 2020). However, it is important to note that IBD does not solely arise from increased gut permeability, but rather results from an interplay of multiple risk factors such as genetics, intestinal microbiota, diet, immune system, and environment (Tamburini et al., 2022; Noble et al., 2023). Over the last half century, the contribution of the “Westernized” diet has become a major concern pertaining to IBD, as urbanization introduced dietary patterns predominantly characterized by high sugar and fat content, frequented antibiotic use, and increased exposure to pollution. Evidently, these factors lead to reduced microbial biodiversity and heightened risk of IBD (Halfvarson et al., 2017; Vich Vila et al., 2018; Pittayanon et al., 2020; Pisani et al., 2022; Adam et al., 2023). While it has been shown that increased gut permeability precedes IBD onset, it is important to note that chronic inflammation can exacerbate intestinal barrier impairment. It is thought that the shift in microflora triggers and maintains inflammation, leading to the chronicity of the disease. Consequently, the inefficiency to dampen inflammation leads to tissue damage and gut permeability (Yacyshyn and Meddings, 1995; Suenaert et al., 2002). Compelling evidence supports the notion that resolving inflammation not only mitigates gut permeability but also ameliorates colitis (Suenaert et al., 2002; Marini et al., 2003; Arrieta et al., 2009). As such addressing the mechanisms that drive intestinal inflammation is essential in order to treat gut hyperpermeability.

## Structure of the gastrointestinal tract and barrier

The gastrointestinal tract is composed of the oral cavity, esophagus, stomach, small intestine, large intestine, and anal cavity. Lining these cavities is a thick mucosal barrier that separates environmental factors from the host tissues (Vancamelbeke and Vermeire, 2017). This semipermeable barrier regulates the absorption of nutrients yet limits the entry of harmful substances into the tissues. The function of the intestinal barrier is controlled by cellular, and chemical components including dietary molecules, microorganisms, immune cells, tissue cells and secreted mucus and antimicrobials (Okumura and Takeda, 2018). Interactions between the gut microbiota, immune system and intestinal cells occur at the mucosal barrier interface, which later shapes host health and disease.

There are over 10 trillion microbes located in the gut lumen that are essential for nutrient breakdown (Joller et al., 2014). These microbes form intricate biochemical niches influenced by various external factors, including lifestyle and diet. To protect against potential harm, intestinal epithelial cells (IECs) establish a mucosal barrier composed of a thick layer of mucus to separate the host immune cells from the gut microbiota (Beck et al., 2006). Another line of host defense against microorganisms are intestinal immune cells such as dendritic cells, macrophages, natural killer cells, B and T cells, that reside at the mucosal surfaces, lamina propria, and immune-enriched follicles (Lord et al., 2010; Gibney et al., 2015). Several factors govern the health of the epithelial barrier, namely dietary molecules, the microbiome, and inflammation. As these factors are interdependent, perturbations to any one of these systems could disrupt the integrity of the gut barrier. T regulatory cells (Tregs) are enriched in the mucosal layer to control inflammation caused by immune-bacterial interactions at the epithelial surface (Atarashi et al., 2011). These cells exert suppressive effects on adaptive and innate immune cells by limiting their differentiation, proliferation, effector functions, and initiating programmed cell death (Sojka et al., 2008). Given the profound impact of inflammation on the epithelial barrier integrity, Tregs serve as a vital link connecting diet, gut microbiota, and host barrier integrity (Arpaia et al., 2013).

### Immune dysregulation in IBD

Approximately 70–80% of the body's immune population is localized within the gastrointestinal tract (Johnson, 1987; Furness et al., 2014). The mucosa and lamina propria contain concentrated clusters of various immune cell types, including dendritic cells (DCs), innate lymphocyte cells (ILCs), intra-epithelial lymphocytes (IELs), macrophages, and T and B cells (Lee et al., 1985; Yuan and Walker, 2004; Zheng et al., 2020). Intestinal immune cells collectively form a highly functional and efficient immunological barrier against bacterial invasion by regulating the integrity and permeability of the intestinal barrier. It is intimately involved with gut microbiota and together they co-evolve to protect the host from external dangers. One example is the strengthening of the mucosal barrier via cytokines, whereby commensal microbes stimulate innate lymphoid cells to produce IL-22. This leads to increases in lipid absorption by epithelial cells and improved barrier function (Talbot et al., 2020). However, in the initial stages of IBD, the compromised intestinal barrier allows for the excessive entry of microbial antigens into the tissue. These antigens trigger the activation of innate immune cells, including neutrophils, DCs, and macrophages. However, prolonged immune activation and impaired bacteria clearance leads to excessive accumulation of neutrophils within the tissue of IBD patients (Vespa et al., 2022). Such occurrences could be attributed to genetic impairments in autophagy observed in IBD patients, whereby impairment autophagy interferes with Paneth and goblet cells function and morphology, cytokine secretion by macrophages, antigen presentation by dendritic cells and epithelial cell stress response (Saitoh et al., 2008; Cooney et al., 2010; Kaser and Blumberg, 2011; Lassen et al., 2014; Iida et al., 2017). The resulting production of inflammatory mediators, which are meant to eliminate bacteria, ends up damaging the host gut tissue, further facilitating bacterial invasion, and prolonging the inflammatory response. In the later stages of the disease, there is a notable activation and expansion of adaptive immune cells, particularly conventional T-helper (Th) 1 and Th17 cells, as they are crucial for combating pathogenic bacteria (Fujino et al., 2003; Cao et al., 2023). These cells primarily secrete interleukin (IL)-17 and interferon (IFN)-γ, which play significant roles in inflammation regulation. One of the key characteristics of IBD is the apparent loss of anti-inflammatory mechanisms, specifically Tregs (Maul et al., 2005; Saruta et al., 2007; Wang et al., 2011; Smids et al., 2018). Tregs represent a distinct subset of CD4^+^ T cells distinguished by their expression of the transcription factor forkhead box P3 (Foxp3), CD25 and low expression of CD127 (Liu et al., 2006). Intestinal Tregs can be further classified into subsets such as Tr1 (IL-10^+^ Foxp3^+^) Tregs, Tr17 (retinoic acid-related orphan receptor-γt [RORγt]^+^Foxp3^+^) Tregs, ICOS^+^ Tregs, Neuropilin-1 (Nrp1^+^GATA-binding protein 3 [GATA3]^+^Helios^+^) Tregs and CD8^+^ Tregs (Figliuolo da Paz et al., 2021). These cells are responsible for maintaining peripheral tolerance by secreting granzymes, perforins, and cytokines such as IL-10, IL-35, and transforming growth factor-β (TGFβ) (Vignali et al., 2008). They constitutively express immunoregulatory proteins such as T cell immunoreceptor with Ig and ITIM domains (TIGIT), cytotoxic T-lymphocyte-associated protein 4 (CTLA4) and programmed cell death protein-1 (PD-1), whereby engagement of the proteins with costimulatory molecules present on antigen presenting cells and T effector cell, leads to effector T cells inactivation and anergy (Levin et al., 2011; Bin Dhuban et al., 2015; Lord, 2015; Tan et al., 2020; Hong and Maleki Vareki, 2022).

Numerous studies have emphasized the crucial role of Tregs in the pathogenesis and progression of colitis. Depletion of Tregs in animals has been observed to spontaneously induce colitis (Rubtsov et al., 2008). Administration of IL-2, a vital factor for Treg proliferation, has shown significant protective effects in preclinical models of IBD, with modest results in patients (Goettel et al., 2019; Allegretti et al., 2021). Similarly, preferential expansion of Tregs via rapamycin prevented colitis development in a CD4 T cell transfer model in mice (Ogino et al., 2012). The cytokines IL-10, IL-35 and TGF-β produced by Tregs are essential in limiting the responses of CD4^+^ T effector cells that drive the progression of IBD. Conditional knock-out of IL-10 in Tregs does not result in systemic autoimmunity but leads to inflammation specifically in the lungs and colon, whereby the unchecked immune response in the colon manifests as spontaneous colitis (Rubtsov et al., 2008). Similarly, mice lacking IL-35 expression in Tregs are unable to limit inflammation in a T cell transfer model of colitis (Collison et al., 2007). The actions of IL-10 from Tregs effectively restrict the development of colitis through specific suppression of Th17 cells, rather than Th1 and Th2 cells (Chaudhry et al., 2011). Whereas TGF-β produced by Tregs have been shown restrain Th1-mediated colitis (Powrie et al., 1996), while overexpression of IL-35 inhibits both Th1 and Th17 responses and facilitates mucosal healing in colitis (Wirtz et al., 2011). It has been shown that TIGIT expression by Tregs selectively inhibits Th1 and Th17 responses but not Th2 responses (Joller et al., 2014). Whilst immunotherapies involving the blockade of CTLA-4 and PD-1 have been associated with the development of enterocolitis (Beck et al., 2006; Brahmer et al., 2010; Lord et al., 2010; Gibney et al., 2015; Dahl et al., 2022). This illustrates the importance of Tregs in regulating Th cell responses and preventing colitis.

There are paradoxical findings surrounding the Treg population and function in individuals with IBD and it is possible that these discrepancies are due to the stage of disease or the specific T cell population under investigation. In the peripheral circulation, Treg numbers tend to decrease during active disease and return to baseline levels upon remission (Maul et al., 2005; Saruta et al., 2007; Wang et al., 2011; Smids et al., 2018). It is likely the peripheral Tregs migrate to the inflamed intestines as evidenced by their accumulation in the mucosal tissue of IBD patients (Maul et al., 2005; Saruta et al., 2007; Wang et al., 2011; Smids et al., 2018). Although Foxp3^+^ Treg populations within the inflamed intestinal mucosa of IBD patients maintain their activation markers and expressions of CTLA4 and PD-1, there are defects in their ability to migrate and repopulate the intestines (Maul et al., 2005; Goldberg et al., 2019). Even though they retain their activation markers, these Tregs become anergic and do not adequately suppress inflammation in IBD (Saruta et al., 2007; Lord et al., 2015). An additional contributing factor of the insufficiency of Tregs in IBD, could be due to T effector cell resistance to Treg-mediated suppression (Fantini et al., 2009).

Naturally, Treg suppression of innate and adaptive immune cells is important in attenuating immunopathology. However, in the context of pathogenic infections, T effector cells evade Treg-mediated suppression to maintain effective immune responses against pathogens whilst innate immune cells are generally suppressed (Freeman et al., 2014). In cases of infection, toll-like receptor (TLR) on T cells are activated leading to the production of IL-6 and TNF-α and this promotes their resistance to Treg-mediated suppression (Pasare and Medzhitov, 2003). The production of these cytokines by T cells alone is insufficient to confer resistance to suppression, indicating the importance of TLR activation by bacteria in mediating resistance against suppression. This would be important in cases of bacterial infection so that the host is able to mount a sufficient immune response. However, in IBD, there is prolonged exposure of the microbiome to inflammatory T cells due to breaches in the gut barrier, further promoting the expansion of T effector cells and production of inflammatory cytokines. In a recent study, the proliferation of Tregs and elimination of microbiota-specific CD4^+^ T cell activation through metabolic checkpoint inhibition protected against colitis (Zhao et al., 2020). As such, resolving the balance of inflammatory and anti-inflammatory T cells, as well as the reparation of the intestinal barrier are essential in limiting chronic inflammatory responses to prevent further tissue damage.

## Change in the microbial landscape after IBD

The gut microbiota demonstrates a high degree of adaptability and can be modified by dietary interventions, thereby offering a potential avenue for therapeutic manipulation. However, this adaptability also presents a risk, as an imbalanced or unhealthy diet can lead to detrimental alterations in the microbiota, rendering the individual more susceptible to disease. Certain bacterial strains, such as *Bacteroides fragilis, Akkermansia muciniphila, Lactobacillus plantarum, Bacteroides thetaiotaomicron*, and *Faecalibacterium prausnitzii*, have been identified to promote gut barrier function, dampen inflammation via Treg activation and enhance the expression of tight junction proteins in IECs (Lavasani et al., 2010; Round et al., 2011; Martín et al., 2015; Wang et al., 2018). Disruptions to these microbial communities, known as gut microbial dysbiosis, have been linked to dysregulations in the immune system, metabolism, and gut hormones (Wu and Wu, 2012). These dysregulations can ultimately contribute to the development of inflammatory and autoimmune diseases, such as colitis (Roy et al., 2017).

The beneficial effects of high-fiber diets have been explored as a potential therapeutic strategy for managing IBD. Dietary fiber consists of indigestible carbohydrates naturally found in plant-based foods. Human digestive enzymes cannot break down certain carbohydrates, but they can be fermented by the gut microbiota. The most extensively studied metabolites are short-chain fatty acids (SCFAs), which include acetate, propionate, and butyrate (Dai and Chau, 2017). High-fiber diets have demonstrated multiple beneficial effects in the context of IBD. They promote the growth of beneficial bacteria, enhance microbial diversity, improve gut barrier function, and exert anti-inflammatory effects through the production of SCFAs (Yusuf et al., 2022). IBD is associated with a westernized diet characterized by decreased fiber intake and increased consumption of sugars and fats (Li et al., 2020). This leads to significant shifts in the composition of the microbiome favoring the growth of *Escherichia coli* and *Fusobacterium* and reductions in beneficial bacteria such as *A. muciniphila* or specifically within the *Clostridium* clusters IV and XIVa, including *F. prausnitzii, Roseburia* species, and *Eubacterium rectale* (Frank et al., 2007; Louis and Flint, 2009; Ohkusa et al., 2009; Sokol et al., 2009; Smith et al., 2013; Zhang et al., 2017; Zhu et al., 2018; Earley et al., 2019; Dubinsky et al., 2022; Liu et al., 2022a). Together, the gut microbiota and dietary molecules play a crucial role in maintaining the population and function of intestinal Tregs through mechanisms such as TLR activation on Tregs or by recognition of bacterial antigens via its T cell receptor (Lathrop et al., 2011; Round et al., 2011). As such, significant shifts in microbial diversity can pose consequences on the individual's ability to resolve inflammation. By addressing the mechanisms that drive post-IBD gut hyperpermeability, the exposure to bacterial antigens, inflammation and tissue damage can be reduced.

### Breakdown of the mucus barrier in IBD

Unlike other organs, the intestinal immune system is constantly exposed to bacterial and foreign antigens. Goblet cells are responsible for producing a thick layer of mucus that acts as a physical barrier separating the microbial-rich lumen from the host tissue and immune cells (Pelaseyed et al., 2014). This mucus layer is predominantly composed of the mucin glycoprotein MUC2, along with secretory immunoglobulin A (IgA) and antimicrobials (Peterson et al., 2007; Meyer-Hoffert et al., 2008; Johansson et al., 2013). Mucosal Tregs regulate the integrity of this barrier by dampening inflammation to auto-antigens and facilitate oral tolerance, both of which are important for the direct health of IECs (Cosovanu and Neumann, 2020). Experimental evidence has illustrated the bidirectional relationship between the mucus barrier and intestinal inflammation (Van der Sluis et al., 2006; Gersemann et al., 2009; Shan et al., 2013; Allenspach et al., 2018; CuŽić et al., 2021). Genetically modified mice deficient in *Muc2* spontaneously develop colitis-like symptoms such as loose stools, diarrhea, and occult (Van der Sluis et al., 2006). These mice also exhibit elevated Th1 and Th17 cells and fewer Tregs in the lamina propria (Shan et al., 2013). In both patients, rodent and canine models of IBD, the destruction of the intestinal tissue leads to the loss of cells in the crypts and goblet cells (Gersemann et al., 2009; Allenspach et al., 2018; CuŽić et al., 2021). Even though chronic UC patients show increased expression of secretory markers and MUC2-positive goblet cells in the intestinal tissue, these cells were unable to produce mucin upon stimulation (van der Post et al., 2019; Singh et al., 2022). This may be due to mucin protein misfolding seen in IBD (Heazlewood et al., 2008). Thus, the mucus layer in IBD is not only thinner, but also altered in function and mucin protein misfolding contributes further to spontaneous inflammation caused by ER stress (Pullan et al., 1994; Heazlewood et al., 2008; Strugala et al., 2008; Braun et al., 2009; van der Post et al., 2019; Kramer et al., 2023).

There is also a greater expansion of mucolytic bacterial strains such as *Ruminococcus gnavus* and *Ruminococcus torques* in the gut mucosae of UC and CD patients, potentially contributing to thinning of the mucus layer (Png et al., 2010; Hall et al., 2017). The thinner mucus barrier makes the epithelial tissue more accessible to commensal bacteria, as rectal biopsies from IBD patients exhibit a greater number of bacteria residing within the mucus layer when compared to healthy individuals (Schultsz et al., 1999). This leads to increased exposure of pathogen-associated molecular patterns (PAMPs) to the immune system, chronic inflammation, and the loss of tolerance to commensal bacteria. In patients with IBD, there were seroreactive antibodies to *Escherichia coli, Pseudomonas fluorescens, Saccharomyces cerevisiae*, and neutrophilic antigens (Landers et al., 2002). Accordingly, there were changes to the relative abundances of *Enterobacteriaceae, Proteobacteria*, and *S. cerevisiae* in the gut microbiota of individuals with IBD (Kaakoush et al., 2012; Sokol et al., 2017; Khorsand et al., 2022). Although experimental studies that have explored the use of oral tolerance to antigens as a form of therapy in IBD have shown success in increasing Treg numbers and reducing colitis, this efficacy has not translated into clinical settings (Kraus et al., 2004; Ino et al., 2016; Paiatto et al., 2017). The authors argue that the defective oral tolerance mechanisms in IBD individuals may be attributed to genetic abnormalities, rather than a specific neoantigen (Kraus et al., 2004).

### Breakdown of the epithelial barrier in IBD

Beneath the mucus layer, is a physical barrier composed of specialized IECs (Boudry et al., 2004). Serving as the interface between the luminal environment and the body, this barrier must be adaptable to the ever-changing intestinal environment during digestion. Intestinal stem cells located in the basal crypts regularly proliferate and differentiate into specialized IECs that replenish the villus tip (Creamer et al., 1961; Umar, 2010). These specialized IECs include the anti-microbial producing enterocytes, mucus-producing goblet cells, secretory Paneth cells, enteroendocrine cells that monitor and regulate intestinal activity during digestion, and M cells that sample foreign antigens in close proximity to lymphoid tissues (Hooper, 2015). Nutrient exchange can occur via passage through the cells or in-between cells, termed transcellular, or paracellular transport, respectively (Edelblum and Turner, 2015).

Paracellular permeability is regulated by the rearrangement of tight junction proteins (TJPs), adherens and gap junctional proteins (Shah and Misra, 2011; Yu and Li, 2014). TJPs are located closest to the lumen and mainly consist of claudins, occludin, and zona occludens (ZO) proteins (Ulluwishewa et al., 2011). Below that are adherens and gap junctions, such as catenins and cadherins, involved with intracellular communication and cell-cell adhesion (Farquhar and Palade, 1963). Redistribution of these paracellular transport constituents can be observed in IBD (Landy et al., 2016; CuŽić et al., 2021; Hu et al., 2021). In dogs with idiopathic IBD, there are no changes to the expression of claudin and β-catenin proteins, but a reduction in E-cadherin (Ohta et al., 2014). Distribution of claudin-2 was varied throughout the colonic intestinal crypts of IBD patients and there was decreased claudin expression adjacent to the mucosal ulcerations, erosions and at sites of neutrophil infiltration (CuŽić et al., 2021). TJP rearrangement was accompanied with augmented epithelial cell proliferation and cell differentiation, and there he was also a loss of tricellulin, a tricellular TJP, in active UC and was restored during remission (CuŽić et al., 2021; Hu et al., 2021). Altered claudin expression in the colon was replicated in mouse models of IBD, with increased expression of claudin-8 in the IECs and claudin-2 in the crypt proliferative zones following dextran sodium sulfate (DSS)-induced colitis (CuŽić et al., 2021). It is conceivable that the loss of TJP in IBD is a consequence of inflammation and rearrangement of IECs to accommodate the influx of immune cells.

## T regulatory cell influence on the mucus barrier

Recent evidence has shown that microbial dysbiosis can disrupt the function of mucosal DCs and disrupt oral tolerance (Fukaya et al., 2023). In the case of IBD, it can be argued that rather than being presented by tolerogenic DCs to Tregs, the damaged epithelium allows luminal antigens enter the host tissue to directly activate immune cells. This highlights the importance of regulated antigen exposure to the mucosal immune system and the role of the mucus barrier. In *Muc2*^−/−^ mice, which lack an intact mucus barrier, there was difficulty in inducing oral tolerance, and this was only restored following the reintroduction of MUC2 into the body (Shan et al., 2013). Ingestion of bacterium coated in MUC2 by DCs mitigates their inflammatory response and induced IL-10 production (Shan et al., 2013). These tolerized DCs inhibited Th1 and Th17 cell proliferation, while promoting Treg Foxp3 expression and expansion (Shan et al., 2013; Parrish et al., 2022). In turn, Treg-derived IL-10 maintain the mucosal immune homeostasis by restraining IL-17-producing cells and lamina propria lymphocytes which contribute to tissue destruction in IBD (Chaudhry et al., 2011; James et al., 2016; Globig et al., 2022). The direct interactions between Tregs and goblet cells may also play a crucial role in maintaining mucosal integrity in IBD. Animals deficient in IL-10 characteristically develop spontaneous colitis and these animals also display a diminished goblet cell population and mucin secretion (Xue et al., 2016; López Cauce et al., 2020; López-Cauce et al., 2023). As mentioned above, one of the issues with the mucus barrier in IBD patients is the phenomenon of mucin protein misfolding and a study done in an animal model of spontaneous colitis revealed that IL-10 administration can preserve mucin protein folding and mucus secretion by goblet cells (Hasnain et al., 2013). As such, establishing a robust population of mucosal Tregs may be key in restoring the gut barrier, regulating antigen presentation, and limiting inflammation.

## T regulatory cell influence on the epithelial barrier

Tregs have been found to promote the integrity of tight junctions, which are crucial for maintaining the barrier function of the intestinal epithelium. Adoptive transfer of Tregs into *Rag1* ^−/−^ mice protected against gut permeability in experimental cirrhosis through the restoration of ZO-1, occludin, claudin 1, and claudin 2 expression (Juanola et al., 2018). In canines with idiopathic IBD, treatment with the probiotic cocktail containing *Lactobacillus, Bifidobacterium*, and *Streptococcus sulivarius* significantly reduced clinical symptoms by reducing CD4^+^ cell infiltrate, increasing the population of Foxp3^+^ Tregs, and increasing occludin and claudin 2 expressions in the intestinal mucosal tissue (Rossi et al., 2014). Administration with a probiotic mixture containing *Bifidobacterium, Lactobacillus acidophilus*, and *Enterococcus* showed success against experimental and clinical IBD, which was attributed to the restoration of the TJP structure, upregulation of colonic Tregs and reduction of colonic TNF-α, IFN-γ, and CD4^+^ cells (Cui et al., 2004; Zhao et al., 2013; Zhang et al., 2018). It is likely that Tregs act to reduce inflammation which alleviates the inflammation-mediated rearrangement of TJPs.

Foxp3^+^ Tregs preserve the balance and stability of IECs by supporting the renewal of epithelial stem cells. *In vitro* organoid studies have demonstrated that Treg cells produce IL-10 to promote of stem cell renewal (Biton et al., 2018). As mentioned above, IL-10 production by Tregs limits tissue pathology and inflammation at the mucosal surface of the colon (Rubtsov et al., 2008). One mechanism by which it does this is by reducing the susceptibility of IECs to inflammatory mediators such as TNF-α and IFN-γ, as well as to T cell-mediated apoptosis via the Fas/Fas ligand (Bharhani et al., 2006). Treg depletion *in vivo* has been linked to reduced proportions of intestinal stem cells and an increase in the rate of IEC differentiation (Biton et al., 2018). Additionally, *IL-10*^−/−^mice display reduced numbers of specialized IECs such as Paneth cells and the diminished population of these cells observed in IBD can impair the host anti-microbial defense against pathogens, nutrient absorption and alter microbial compositions (Schopf et al., 2002; Simmonds et al., 2014; Xue et al., 2016; Shimizu et al., 2020; Wehkamp and Stange, 2020).

Signals sent from IEC also influence Treg population and function. IEC-derived factors such as TGF-β and retinoic acid induce a tolerogenic phenotype in DCs, leading to the differentiation of Tregs capable of protecting against colitis (Iliev et al., 2009). Compared to Tregs found within the lymph nodes, Foxp3^+^ Tregs residing with the epithelial barrier lose the requirement for IL-2 to survive, have reduced CD25 expression and upregulate CTLA4, thereby enhancing their suppressive capabilities (Prakhar et al., 2021). Maintenance of the IEC population is essential as apoptotic IECs leads to the loss of Foxp3^+^ Treg cells (Nakahashi-Oda et al., 2016). As such, the destroyed tissue barrier and reduction of IECs in IBD would inevitably have negative consequences on the intestinal Treg population and anti-inflammatory immune response.

### Nutrition, the microbiome, and T regulatory cell interactions

**Figure 1:**
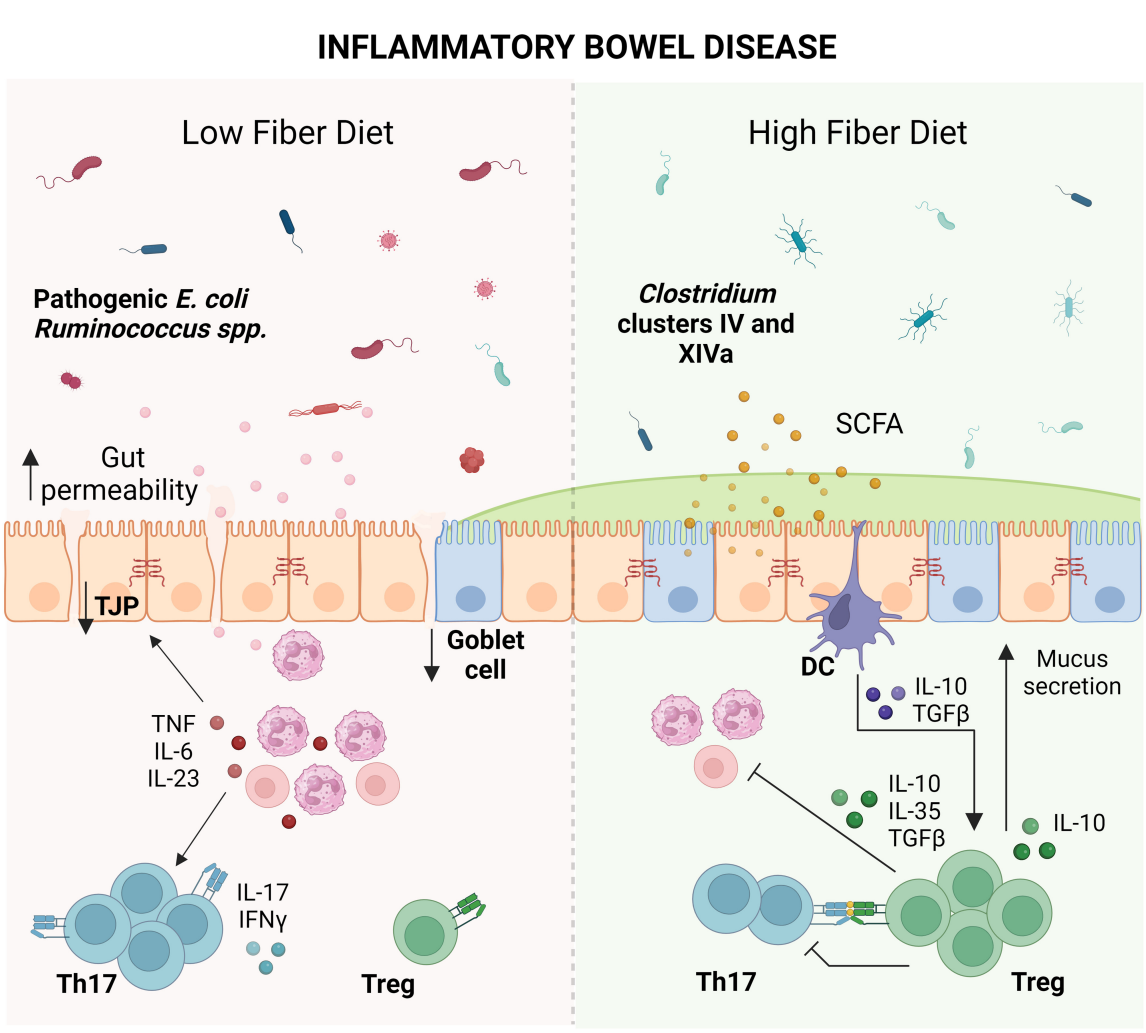
Impact of dietary fiber on inflammatory bowel disease (IBD). IBD coupled with a low fiber diet leads to microbial dysbiosis. Microbial antigens released into the intestinal milieu are recognized by immune cells, leading to immune activation and recruitment of T cells such as T helper (Th) 17 cells. Overproduction of inflammatory cytokines such as tumor necrosis factor (TNF), interleukin (IL)-6, IL-23, IL-17, and interferon (IFN)-γ damages the epithelial barrier, leading to tight junction protein (TJP) loss, goblet cell loss, decreased mucus production and increased gut permeability. Elevated gut permeability perpetuates microbial exposure and immune activation, leading to chronic inflammation and tissue damage. Conversely, consumption of dietary fiber leads to the expansion of short-chain fatty acid (SCFA)-producing bacteria from the *Clostridium* clusters IV and XIVa. SCFAs strengthen the gut barrier by serving as fuel to colonocytes and tolerize dendritic cells (DCs), leading to production of anti-inflammatory cytokines such as IL-10 and transforming growth factor-β (TGFβ). These cytokines promote the growth of T regulatory cells (Tregs) which dampen inflammatory cells as well as promote goblet cell function and mucus secretion, providing an opportunity for reconstitution of the epithelial barrier (Created with BioRender.com).

Effective IBD therapy needs to be able to concomitantly address the breakdown in the microbial populations, gut permeability, mucus barrier, and Treg populations. Given the association between microbial dysbiosis and chronic intestinal inflammation, it is understood that diet plays a crucial role in the management of disease. Multiple clinical studies of IBD have demonstrated that fiber supplementation helped in reducing inflammatory cytokines, microbial dysbiosis and improving remission rates (Faghfoori et al., 2011, 2014; Chiba et al., 2015; Fritsch et al., 2021). Due to its anti-inflammatory effects, it is recommended that patients slowly introduce fiber back into their diet in tolerable doses. Dietary factors not only directly affect the Treg population but may also exert indirect effects through the gut microbiota (Smith et al., 2013; Geirnaert et al., 2014; Biton et al., 2018; Nie et al., 2021; Yoshimatsu et al., 2022; Bourdeau-Julien et al., 2023). In turn, Tregs can greatly enhance the anti-inflammatory potential of dietary fiber for the treatment of IBD (Gaudier et al., 2004; Pérez-Reytor et al., 2021). IBD-associated microbial dysbiosis that compromises the breakdown of dietary fiber, could significantly influence the individual's capacity to control inflammation (Frank et al., 2007; Louis and Flint, 2009; Sokol et al., 2009; Smith et al., 2013; Zhang et al., 2017; Zhu et al., 2018; Earley et al., 2019; Liu et al., 2022a). As such, the impact of nutrition should be explored for its role in reshaping the population and function of Tregs in IBD in order to restore the intestinal barrier (Figure 1).

Short-term modifications in dietary patterns can have an impact on the composition of gut microbiota. Studies in individuals have demonstrated significant shifts in the bacterial and metabolic profiles involving SCFA-producing bacteria *Butyricicoccus* and *Roseburia* within a period of 3 days following extreme dietary changes (Geirnaert et al., 2014; Nie et al., 2021; Bourdeau-Julien et al., 2023). The gut microbiota can directly modulate Treg differentiation, function, and survival, but they can also have indirect effects via the metabolites they produce. Notably, colonic Tregs have high expressions of the gene encoding G coupled protein receptors (GPRs), and aryl hydrocarbon receptor (AhR) (Smith et al., 2013; Ye et al., 2017; Rothhammer and Quintana, 2019; Jiang and Wu, 2022). This enables Tregs to effectively sense and respond to dietary and microbial metabolites. However, genetic defects in IBD individuals such as in the CARD9 susceptibility gene, can impair their ability to produce AhR agonists (Lamas et al., 2016). This interferes with their ability to appropriately respond to bacterial metabolites and could explain the significant influence of diet on the development of this disorder and the persistence gut barrier disruptions and permeability. Studies have demonstrated that stimulation of AhR can promote the recruitment of colonic Tregs which facilitate the proliferation of intestinal stem cells (Biton et al., 2018; Yoshimatsu et al., 2022). Specifically, dietary changes can alter the population of the *B. thetaiotaomicron* bacterial species, which in turn in is able to activate AhR to promote Treg differentiation (Wegorzewska et al., 2019; Li et al., 2021). Treatment with an AhR ligand have been found to alleviate TNBS colitis by increasing the population of IL-10 secreting Tregs (Goettel Jeremy et al., 2016). However, clinical use of AhR agonists for treating refractory UC has not been widely recommended due to potential adverse effects such as pulmonary arterial hypertension (Naganuma et al., 2018; Yoshimatsu et al., 2019; Saiki et al., 2021).

Given the diverse range of beneficial effects, dietary fiber ease of access, and more importantly, its safety profile, dietary fiber can be highlighted as an important contributor to epithelial barrier integrity and Treg function. Indeed, SCFAs supplementation promotes the expansion of colonic Tregs in germ-free mice and subsequent propionate challenge significantly increases *Foxp3* and *IL-10* expressions in these Tregs (Smith et al., 2013). In turn, Treg treatment can help maintain SCFA concentration in the intestines, which would typically be diminished following bacterial challenge (Juanola et al., 2018). Under homeostatic conditions, mucin-adherent microbiota, including bacterial species from the *Clostridium* cluster XIVa, and *Roseburia intestinalis* and *E. rectale* produce butyrate near the epithelium (Van den Abbeele et al., 2013). The decrease of these microbial species in IBD could potentially be due to the reduction in available mucus for their adhesion. Consequently, this would decrease the bioavailability of SCFAs to IECs and hence compromise the regeneration of the gut barrier. Whilst SCFAs have been described to elicit direct effects on goblet cells and IECs to improve mucus secretion and epithelial health, it is insufficient to downregulate the overwhelming inflammatory status of the intestines without the aid of Tregs (Gaudier et al., 2004; Pérez-Reytor et al., 2021). In an animal model of gut inflammation caused by *Candida albicans*, treatment with SCFAs in Treg-depleted mice could not resolve inflammation alone, but found that SCFAs induced Foxp3^+^ Tregs which mediated protective effects during mucosal inflammation (Bhaskaran et al., 2018). Similarly, in a T cell transfer model of colitis, mice lacking in T and B cells were treated with SFCA mix or propionate in the absence of Tregs. They showed no improvement in disease severity, but it was only through the combination of Tregs and SCFAs added to the system that the colitis was healed (Smith et al., 2013). Sun et al., showed that SCFA-treated CD4^+^ T cells produced more IL-10 and lessened the severity of colitis (Sun et al., 2018). These SCFA-mediated effects were diminished following treatment with an anti-IL-10R antibody, further highlighting the importance of immune-related mechanisms for the abrogation of inflammation post-IBD (Sun et al., 2018). These findings highlight the key interplay between bacterial metabolites and Treg activity.

Shifts in the gut microbiota significantly affect the individual's ability to regulate inflammation. IBD is marked by a reduction of SCFA-producing bacteria such as *A. muciniphila* or *Clostridium* species including *F. prausnitzii, Roseburia* species, and *E. rectale* (Frank et al., 2007; Louis and Flint, 2009; Sokol et al., 2009; Smith et al., 2013; Zhang et al., 2017; Zhu et al., 2018; Earley et al., 2019; Liu et al., 2022a). The depletion of SCFA-producing microbial populations has detrimental effects on the ability of the intestinal immune system to regulate inflammation. These SCFA-producing bacterial strains play a crucial role in maintaining the population of colonic Foxp3^+^ Tregs for the protection against colitis (Atarashi et al., 2011). More specifically, *A. muciniphila* supplementation has been proven beneficial against CD4 T cell transfer model of colitis in animals through the upregulation of RORγt^+^Foxp3^+^ T regulatory 17 cells (Liu et al., 2022b). These RORγt^+^Foxp3^+^ Tregs also express immunoregulatory markers and can effectively suppress intestinal inflammation (Yang et al., 2016). Colonization by commensal bacterial species from the *Clostridium* clusters IV and XIVa promotes the differentiation and accumulation of Tregs in the mucosal tissues of mice with DSS-induced colitis (Atarashi et al., 2011; Smith et al., 2013). Colonization by *E. rectale* facilitates Treg differentiation and function (Islam et al., 2021). Both *F. prausnitzii* and *R. intestinalis* have demonstrated the ability to increase Treg populations and alleviate 2,4,6-trinitrobenzenesulfonic acid (TNBS)-induced colitis in animals (Qiu et al., 2013; Zhu et al., 2018). The bacteria not only enhance the population of Tregs, but also their functions via their metabolites. It was shown that Tregs from individuals treated with butyrate showed increased secretion of IL-10 and adoptive transfer of butyrate-induced Tregs possess the ability to ameliorate colitis in mice (Furusawa et al., 2013; Mamontov et al., 2015). Apart from its influence on Tregs, the bacteria *F. prausnitzii* can promote IL-10 and TGF-β secretion by human peripheral blood mononuclear cells (PBMCs), while *R. intestinalis* inhibits the LPS-induced production of IL-17 by human colonocytes (Qiu et al., 2013; Zhu et al., 2018). As such, the loss of these microbial species in IBD may contribute to the excessive inflammatory responses and the impaired ability of Tregs to suppress such inflammation.

Current studies exploring the use of Tregs for the treatment of IBD has shown promise. Preclinical data have demonstrated the efficacy of these cells using *in vivo* models of colitis (Ogino et al., 2012; Goettel et al., 2019). With similar beneficial findings seen in a phase 1b/2a clinical trial involving infusion with low doses of the Treg growth factor IL-2 (Allegretti et al., 2021). Allegretti et al. (2021), trialed several doses of IL-2, with the lowest dose successfully leading to peripheral Treg expansion, but also activation of T effector cells. Upon increasing IL-2 dose and Treg population, over 38% of patients were able to achieve a clinical response or remission. Direct treatment with autologous Tregs was tested in a 1/2a clinical trial involving patients with CD and in this study, Treg treatment was well tolerated and elicited beneficial effects, albeit transient (Desreumaux et al., 2012). This may be due to issues with phenotypic stability and initial screening of the cells used for treatment. As such, there are still unanswered questions to the expansion, homing properties, site-specific activation, survival, and phenotypic stability of Tregs that need to be assessed clinically. This would be important to guarantee the safety and efficacy of Tregs as a form of treatment. Overall, Treg-focused therapies may serve as a promising approach for treating IBD, with more research needed to determine the optimal treatment protocol.

## Conclusion

Regulatory T cells play a central role in maintaining gut barrier integrity. Reciprocal interactions occur between the host microbiota, dietary molecules, and T regulatory cells for the preservation of oral tolerance, the mucus barrier, epithelial cells, and regulated immune responses. They prevent excessive inflammation characteristic of IBD and can therefore protect the gut barrier from ongoing inflammatory damage. By understanding the interplay between nutrition, the microbiome, and its impact on Tregs for the regulation of the intestinal barrier, one can better address the mechanisms that drive IBD pathogenesis.

## Author contributions

KP: Conceptualization, Investigation, Writing – original draft, Writing – review & editing. JO: Writing – review & editing, Funding acquisition. RG: Conceptualization, Investigation, Writing – review & editing.
